# Urinary urodilatin levels in patients with renal salt wasting syndrome as a possible new diagnostic marker. A pilot study

**DOI:** 10.3389/fphar.2025.1579699

**Published:** 2025-06-11

**Authors:** Vittoriano Della Corte, Luisa Agnello, Rosario Norrito, Marco Cataldi, Fabio Del Ben, Rosaria Pecoraro, Carlo Maida, Caterina Maria Gambino, Mario Daidone, Rosaria Vincenza Giglio, Marcello Ciaccio, Antonino Tuttolomondo

**Affiliations:** ^1^ Internal Medicine and Stroke Care Ward, University Hospital “P. Giaccone”, Palermo, Italy; ^2^ Department of Health Promotion, Mother and Child Care, Internal Medicine and Medical Specialties (ProMISE), University of Palermo, Palermo, Italy; ^3^ Clinical Molecular Medicine and Clinical Laboratory Medicine, Department of Biomedicine, Neurosciences and Advanced Diagnostics, Institute of Clinical Biochemistry, University of Palermo, Palermo, Italy; ^4^ Immunopathology and Cancer Biomarkers Unit, Department of Cancer Research and Advanced Diagnostics, CRO Aviano National Cancer Institute IRCCS, Aviano, Italy; ^5^ Department of Laboratory Medicine, University Hospital “P. Giaccone”, Palermo, Italy; ^6^ PhD programme of Molecular and Clinical Medicine, University of Palermo, Palermo, Italy

**Keywords:** renal salt wasting syndrome, urodilatin, ANP, natriuretic peptide, hyponatremia

## Abstract

**Background:**

Renal Salt Wasting Syndrome (RSW) is a clinical syndrome with laboratory characteristics completely overlapping with the syndrome of inappropriate antidiuretic hormone secretion (SIADH). No studies have yet investigated the potential role of urodilatin as a diagnostic marker or its involvement in the pathogenesis of RSW.

**Methods:**

We performed a retrospective observational case-control study, the patients were divided into 3 groups: a group of hyponatremic patients with RSW (cases) and two control groups (subjects without hyponatremia and subjects with hyponatremia from other causes). Main outcomes were assessing the differences in urinary urodilatin values in patients with RSW compared to both control groups and to evaluate the diagnostic power of urodilatin with the analysis of ROC curves.

**Results:**

Patients with RSW display significantly higher mean urodilatin levels than both patients with (median 5.46 vs. 0.57 ng/mL, p = 0.006) or without hyponatremia (median 5.46 vs. 0.27 ng/mL, p < 0.001). Diagnostics performances of mean urodilatin levels for RSW diagnosis were evaluated by ROC curve, AUC was 0.94 (95%CI 0.86–1.00).

**Conclusion:**

This case-control study has shown interesting results regarding the dosage of urinary urodilatin in patients with RSW, with potentially clarifying implications both regarding the pathogenesis of this syndrome and regarding the diagnosis and therefore the clinical management of patients.

## Introduction

Renal Salt Wasting Syndrome (RSW) is a clinical syndrome with laboratory characteristics completely overlapping with the syndrome of inappropriate antidiuretic hormone secretion (SIADH) such as hyponatremia, normal adrenal, thyroid and renal function, concentrated urine with urinary sodium (Una) usually>30 mEq/L and hypouricemia (Maesaka et al., 2020; Assadi and Mazaheri, 2020; Bitew et al., 2009; Schwartz et al., 1957). The fundamental difference between the two syndromes lies in the extracellular volume (ECV) and therefore in the water balance. In fact, in the RSW we will have a reduction of the ECV due to a renal loss of water and sodium with negative water balance, while in the SIADH we will have a normal or slightly increased ECV with a balance substantially in equilibrium (Maesaka et al., 2020; Assadi and Mazaheri, 2020; Bitew et al., 2009; Schwartz et al., 1957). Consequently we will have the diametrically opposite therapeutic goals of water-restricting patients with SIADH or administering salt and water to patients with RSW (Maesaka et al., 2020; Assadi and Mazaheri, 2020; Bitew et al., 2009; Schwartz et al., 1957). Differentiating extracellular volume (ECV) status in clinical practice is challenging, complicating the differential diagnosis of the two syndromes. Furthermore, in SIADH a precise pathogenetic mechanism has been identified together with its protagonist (antidiuretic hormone, ADH) (Schwartz et al., 1957). This has not yet happened for RSW, contributing not only to making diagnosis difficult but also to feed a certain skepticism towards the very existence of the syndrome by some authors (Maesaka et al., 1999; Oh and Carroll, 1999; Singh et al., 2002; Peters et al., 1950).

Clarifying the pathogenesis of RSW is crucial for establishing it as a recognized cause of hyponatremia, dispelling skepticism, and facilitating diagnosis.

As regards the pathogenetic mechanisms so far called to explain RSW, we can recall an alteration of the renal sympathetic afferents and the involvement of natriuretic peptides (NPs) such as the Atrial natriuretic peptide (ANP) (Cerdà-Esteve et al., 2008; Fukuoka et al., 2017; Bitew et al., 2009; Maesaka et al., 2007; Maesaka et al., 2009; Oh and Shin, 2015; Youmans et al., 2013; Della Corte et al., 2018; DiBona, 2000).

### Urodilatin

Urodilatin (URO) is a 32-amino acid peptide belonging to the family of natriuretic peptides first isolated from human urine in 1988 by Forssmann and colleagues. It comprises the amino acid sequence 95–126 of cardiac proANP and shares an identical structure with the circulating 28-amino acid human atrial natriuretic peptide (ANP), except for the addition of four amino acids (Thr-Ala-Pro-Arg) at the NH_2_ terminus (Potter et al., 2009; Lisy et al., 1999; Hirsch et al., 2006; Forte, 2004; Costa et al., 2009; Zhang et al., 2010; Askar and Tarif, 2007; Leonard et al., 2015; Berendes et al., 1997; Kurokawa et al., 1996; Tsubokawa et al., 2003; McGirt et al., 2004; Tomida et al., 1998; Gao et al., 2006; Goetz et al., 1990; Drummer et al., 1996; Della Corte et al., 2023).

Urodilatin (URO) is detectable in human urine but not in plasma, suggesting that it is synthesized and secreted exclusively by the kidneys. It is likely produced in the distal cortical nephron and secreted into the lumen of the kidney tubules, where it exerts paracrine effects. Like atrial natriuretic peptide (ANP), URO binds to natriuretic peptide type A receptors in the inner medullary collecting duct, triggering an increase in intracellular cyclic guanosine monophosphate (cGMP) levels and promoting renal sodium and water excretion (Potter et al., 2009; Lisy et al., 1999; Hirsch et al., 2006; Forte, 2004; Costa et al., 2009; Zhang et al., 2010; Askar and Tarif, 2007; Leonard et al., 2015; Berendes et al., 1997; Kurokawa et al., 1996; Tsubokawa et al., 2003; McGirt et al., 2004; Tomida et al., 1998; Gao et al., 2006; Goetz et al., 1990; Drummer et al., 1996; Della Corte et al., 2023).

After the isolation of the urodilatin natriuretic peptide from human urine, more and more data have been generated supporting the view that in fact not ANP but urodilatin appears to be the natriuretic peptide responsible for renal manipulation of sodium (Goetz et al., 1990; Drummer et al., 1996; Della Corte et al., 2023; Drummer et al., 1993; Herten et al., 1998; Zaid et al., 2007; Anker et al., 2015; Emmeluth et al., 1992; Meyer et al., 1996; Lenz et al., 1999; Forssmann et al., 1998; Maesaka et al., 2021). On the contrary, due to the rapid secretion of ANP as a response to some cardiovascular stimuli and due to numerous effects on the cardiovascular system, it seems reasonable to postulate that the primary target of ANP is the cardiovascular system and not the kidney (Hirsch et al., 2006; Goetz et al., 1990; Della Corte et al., 2023).

Unlike ANP, which is rapidly degraded and inactivated in the kidney by a metalloendoprotease derived from the renal cortex membrane, URO remains active, suggesting that its primary physiological target is the intrarenal ANP-receptor/guanylate cyclase system rather than systemic ANP. However, the mechanisms regulating URO production and excretion are not yet fully understood (Tomida et al., 1998; Gao et al., 2006; Goetz et al., 1990; Drummer et al., 1996; Della Corte et al., 2023).

Studies indicate that renal perfusion pressure, left atrial distension, and cephalic sodium concentration influence URO excretion. Heringlake and colleagues demonstrated that changes in arterial and renal perfusion pressure affect URO release, with experiments in isolated perfused rat kidneys confirming that renal blood flow and pressure are key determinants. Additionally, left atrial elongation has been shown to stimulate URO excretion (Tomida et al., 1998; Gao et al., 2006; Goetz et al., 1990; Drummer et al., 1996; Della Corte et al., 2023).

In conscious dogs, Goetz and colleagues observed that sodium excretion correlated more closely with urinary URO than with plasma ANP, and this effect was abolished in heart-denervated dogs, suggesting a neuronal connection between the heart and kidneys. Further studies using the split infusion technique showed that hypertonic saline infusion into the carotid artery increased URO and sodium excretion, indicating a possible link between cephalic sodium chemoreceptors and renal URO secretion. However, the effect persisted even in denervated kidneys, implying the involvement of an additional humoral factor that transmits brain sodium levels to the kidneys (Tomida et al., 1998; Gao et al., 2006; Goetz et al., 1990; Drummer et al., 1996; Della Corte et al., 2023).

Moreover, URO secretion has been associated with immersion in water in humans, possibly mediated by the renal sympathetic nervous system or dopaminergic nerves. Taken together, these findings suggest that multiple factors regulate URO release, including left atrial distension, sodium concentration, renal perfusion pressure, and neural or hormonal pathways. While the exact initial stimulus remains unclear, extracellular sodium levels appear to play a central role in this regulatory process (Tomida et al., 1998; Gao et al., 2006; Goetz et al., 1990; Drummer et al., 1996; Della Corte et al., 2023).

The kidney seems to have a central role in RSW and no studies have yet been done on the only natriuretic peptide produced in the kidney and with action on the same organ, the urodilatin. Based on the above, we conducted this case-control study to determine the differences in urinary urodilatin levels in patients with RSW (cases) compared to controls and the possible diagnostic power of urodilatin.

## Materials and methods

### Study population

We performed a retrospective observational case-control study at the University hospital “P. Giaccone” of Palermo. We enrolled patients admitted to the Internal Medicine and Stroke Care ward of this hospital.

The patients recruited were divided into 3 groups including a case group and two control groups based on blood sodium values (hyponatraemia was defined as blood sodium values < 135 mEq/L):

• a group of patients without hyponatremia (controls)
• a group of hyponatremic patients with RSW (cases)
• a group of patients with hyponatremia from other causes (controls)

We divided the population this way to verify that differences in urinary urodilatin levels depend on the cause of hyponatremia rather than on reduced plasma sodium levels. The Institutional Ethics Committee of “P. Giaccone” University Hospital approved the study protocol. All procedures performed in studies involving human participants were in accordance with the ethical standards of the institutional and/or national research committee and with the 1964 Helsinki Declaration and its later amendments or comparable ethical standards. Informed consent was obtained from all individual participants included in the study.

### Diagnostic criteria of RSW and patient recruitment

Diagnosis of RSW was made when all the criteria in Table 1 were met. In particular, only patients with polyuria (defined as urine output> 30 mL/kg/day) (Borgmann et al., 2014) not explainable with causes other than RSW (see Table 2) in association with a clearly negative water balance (output - input> 500 mL/day) and a reduction of ECV were included in the RSW group. Selecting this patient population ensured a more accurate diagnosis of RSW. Indeed, since the main diagnostic problem is that of the differential diagnosis with SIADH, these criteria have allowed us to exclude patients with SIADH with reasonable accuracy, who cannot by definition have (given the intrinsic function of ADH which is precisely an antidiuretic hormone) polyuria in the absence of the common causes listed in Table 2. (Schwartz et al., 1957). The assessment of the water balance and the ECV were performed according to the common methods of clinical practice.

**TABLE 1:** RSW diagnostic criteria.

RSW diagnostic criteria
- Hyponatremia: blood sodium <135 mEq/L
- Normal adrenal, thyroid and renal function
- High Una (>40 mEq/L)
- Exclusion of other causes of renal sodium loss (diuretics, acute tubular damage, renal failure…)
- Reduction of the ECV
- Negative water balance (Input – Output < −500 mL/day)
- Polyuria (urine output> 30 mL/kg/day) in the absence of causes other than RSW (see Table 2)

Una, urinary sodium; ECV, extracellular volume.

**TABLE 2:** Causes of polyuria other than RSW.

Causes of polyuria
Aqueous diuresis
Central diabetes insipidus (partial or complete) • Hereditary • Acquired (as a result of injuries, tumors or other injuries) Nephrogenic diabetes insipidus • Amyloidosis • Medicines (lithium, cidofovir, foscarnet) • Hypercalcemia (due to cancer, hyperparathyroidism, or granulomatous disease) • Hereditary diseases • Hypokalaemia • Obstructive Uropathy • Sickle cell disease • Sjögren’s syndrome Polydipsia • Primary (hypothalamic lesions in the thirst center) • Psychogenic Excessive IV hypotonic fluid administration Use of diuretics Adipsic diabetes insipidus Gestational diabetes insipidus
Diuresis of solutes
Uncontrolled diabetes mellitus Isotonic or hypertonic saline infusions High-protein enteral feeding Resolution of urinary tract obstruction

### Biochemical analysis

Routine clinical chemistry parameters were measured for each patient at the hospital admission immediately after sample collection. Urodilatin was evaluated on urine samples collected at 6 a.m. and 2 p.m. in the first 24 h of hospital admission and stored at −80°C until analysis. pro-ANP was assessed on plasma obtained by centrifugation of whole blood sample collected in K3-EDTA tubes upon admission and stored at −80°C until analysis. Serum and urinary biochemical parameters, including alanine aminotransferase (ALT), aspartate aminotransferase (AST), total bilirubin, serum creatinine (sCR), uric acid, glucose, TSH, albumin, cortisol, sodium and potassium, were measured on Cobas^®^ 8000's (Roche, Basel, Switzerland), according to the manufacturer’s procedures. The glomerular filtration rate (GFR) was estimated by was calculated using the Chronic Kidney Disease EPIdemiology collaboration (CKD-EPI) equation expressed for the specified race, gender, and sCR in mg/dL (Levey and Stevens, 2010). Urodilatin and pro-ANP levels were measured by commercially available enzyme linked immunosorbent assay (ELISA) kits, according to the manufacturer’s instructions (BMA Biomedicals, Switzerland and Biomedica Medizinprodukte GmbH, respectively). Hematological tests, including hematocrit, were performed on Sysmex XN-9000 hematology analyzer (Sysmex Corporation, Kobe, Japan). All routine biochemical analyses were performed at the Department of Laboratory Medicine-University Hospital “P. Giaccone” of Palermo, and the measurement of urodilatin and pro-ANP was performed at the Institute of Clinical Biochemistry, Clinical Molecular Medicine and Clinical Laboratory Medicine- University Hospital “P. Giaccone” of Palermo.

### Statistical analysis

Statistical analyses were performed by SPSS statistical software v.17.0 (SPSS Inc., Chicago, IL, United States) and R Language v.4.0.3 (R Foundation for Statistical Computing, Vienna, Austria). Normality distribution was assessed preliminarily by q-q plot and Shapiro–Wilk test. Quantitative variables were expressed by the median and interquartile range (IQR), while qualitative variables by absolute or relative frequency. Differences between groups for continuous variables were estimated by Kruskal–Wallis (>2groups) or Mann-Whitney test (2 groups) with Bonferroni’s correction when needed. Differences between paired groups were studied by nonparametric Wilcoxon Signed Ranks Test. The Correlation was evaluated by the nonparametric Spearman test. Diagnostic accuracy for RSW diagnosis was evaluated by Receiver Operating Characteristic (ROC) curve analysis and reported as Area Under the Curve (AUC) and 95% confidence interval calculated by the DeLong method. The Best cut-off was evaluated by the Youden’s index.

## Results

**FIGURE 1:**
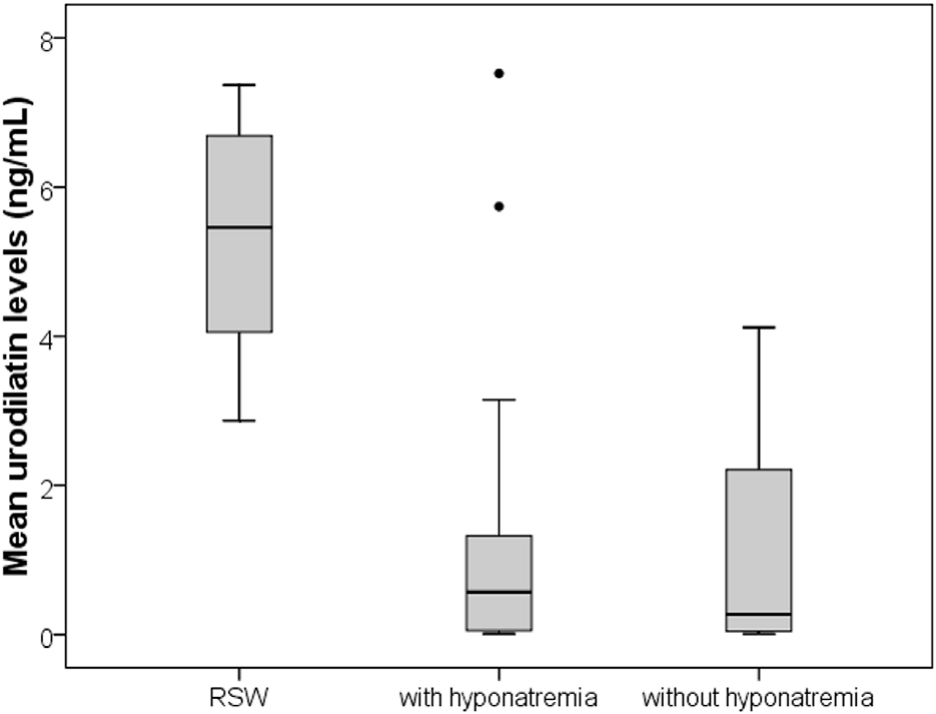
Boxplots comparing mean urinary urodilatin levels among patients with RSW, hyponatremia from other causes, and controls without hyponatremia. RSW patients show significantly higher urodilatin levels (p < 0.001).

**FIGURE 2:**
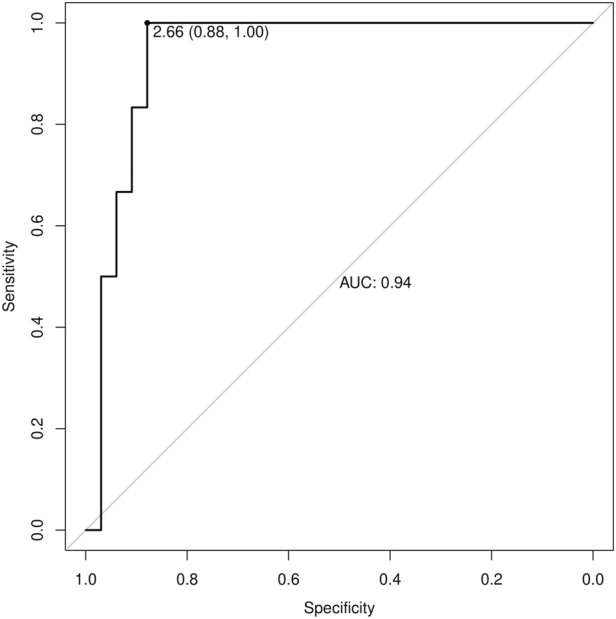
Receiver Operating Characteristic (ROC) curve for urinary urodilatin levels in diagnosing RSW. The AUC is 0.94, indicating excellent diagnostic performance. The optimal cutoff value of 2.87 ng/mL provides 100% sensitivity and 88% specificity.

Thirty-nine subjects were studied. They were sub-grouped, according to previous clinical diagnosis or sodium levels, into patients with Renal Salt Wasting (RSW) syndrome (n = 6 patients with diagnosis made when all the criteria in Table 1 were met), patients with hyponatremia (n = 17 patients with blood sodium values < 135 mEq/L) and patients without hyponatremia (n = 16 patients with blood sodium values ≥ 135 mEq/L). Demographic, clinical and biochemical characteristics of the study population are shown in Table 3. In our study, 23 patients with hyponatremia were recruited. Of these, 6 patients met the diagnostic criteria of RSW, 2 had SIADH, 5 had hyponatraemia related to renal insufficiency (GFR <45 mL/min), 1 case of hypothyroidism, 7 cases of drug-related hyponatremia (use of diuretics predominantly). About the 6 patients with RSW, 3 had intracranial disease (2 with ischemic stroke and 1 with lung cancer brain metastases). The other 3, on the other hand, did not have intracranial pathologies and were hospitalized for the following reasons: 1 case of pneumonia, 1 case of respiratory failure in a patient with lung cancer and 1 case of alterations in consciousness due to hyponatremia. As urodilatin levels measured at 6 a.m. and 2 p.m. were not significantly different (p = 0.05), their mean value was used for subsequent analyses. Interestingly, mean levels of urodilatin were significantly different among the 3 subgroups (overall KW test p = 0.003) (Figure 1). Moreover, taking into account the Bonferroni’s correction, patients with RSW display significantly higher mean urodilatin levels than both patients with (median 5.46 vs. 0.57 ng/mL, p = 0.006) or without hyponatremia (median 5.46 vs. 0.27 ng/mL, p < 0.001) (Figure 1). Statistically significant higher mean levels of urodilatin were also observed when patients with RSW were compared with the other two groups of patients considered together (5.46 vs. 0.32 ng/mL, MW test p < 0.001). Conversely, proANP levels were not statistically different among the 3 subgroups (overall KS test p = 0.266) or between patients with RSW and patients with/without hyponatremia (4.9 vs. 9.7 nM, MW test p = 0.122). In the whole sample investigated, no association was evident between proANP and mean urodilatin levels (Speaman’s rho = 0.059, p = 0.739). However, few correlations were found to be statistically significant when the analysis was performed within subgroups. In particular, in RSW patients proANP was highly inversely correlated with 24 h urine output (rho = −0.975, p = 0.005). Diagnostics performances of mean urodilatin levels for RSW diagnosis were evaluated by ROC curve (Figure 2). Area under the curve (AUC) was 0.94 (95%CI 0.86–1.00). Best cut-off for mean urodilatin levels, according to Youden’s index, was 2.87 ng/mL. At this cut-off sensitivity, specificity, positive predictive value and negative predictive value were, respectively, 1.00, 0.88, 0.60 and 1.00.

**TABLE 3:** Demographic and clinical characteristics of patients in the study, stratified by RSW status and presence of hyponatremia. Data are presented as median (IQR) or frequency (%). Comparisons between groups were performed using the Kruskal–Wallis test (continuous variables) or Chi-square test (categorical variables).

Demographic, Clinical and Biochemical Variables	Patients with RSW (n = 6)	Patients with hyponatremia (n = 17)	Patients without hypoNa (n = 16)	P-value
Demographic				
Sex, M	83%	77%	56%	
Age, years	68 (62–88)	70 (60–77)	72 (64–84)	0.413
Clinical				
Height, cm	170 (168–175)	170 (165–175)	165 (150–170)	0.543
Weight, kg	70 (70–100)	75 (70–82)	78 (64–90)	0.083
BMI, kg/m^2	29.4 (22.9–35.8)	27.7 (22.9–30.1)	28.3 (25.4–31.1)	0.956
PAS, mmHg	120 (110–130)	130 (120–145)	120 (110–133)	0.717
PAD, mmHg	70 (70–85)	75 (70–85)	70 (60–73)	0.141
24 h urine output, mL	3,550 (2,200–5,475)	2,400 (1,250–4,050)	2,400 (1,469–3950)	0.355
Hypertension	2/6	15/17	8/16	0.381
Diabetes	0/6	8/17	6/16	0.010†
Ischemic heart disease	1/6	6/17	6/16	0.127
Heart failure	0/6	2/17	5/16	0.809
Cerebrovascular disease	2/6	2/17	0/16	0.274
COPD	0/6	2/17	4/16	0.077
Atrial fibrillation	1/6	1/17	4/16	0.506
Kidney failure	0/6	7/17	5/16	0.285
Chronic liver disease	1/6	1/17	1/16	0.211
Osteoporosis	0/6	1/17	1/16	0.539
Cancer disease	1/6	3/17	3/16	1.000
Diuretics	0/6	11/17	9/16	0.020
Hyponatremia	6/6	17/17	0/16	<0.001†
Intracranial pathology	3/6	3/17	0/16	0.016
Biochemical				
ProANP, nM	4.9 (1.0–10.3)	9.8 (4.2–13.2)	9.7 (4.9–11.3)	0.266
Urodilatin at 6 a.m., ng/mL	7.04 (3.16–7.55)	0.15 (0.01–1.83)	0.09 (0.01–1.30)	0.007†
Urodilatin at 2 p.m., ng/mL	5.37 (2.69–5.87)	0.77 (0.07–2.06)	0.37 (0.01–2.77)	0.140
Mean urodilatin, ng/mL	5.46 (3.76–6.86)	0.57 (0.05–1.60)	0.27 (0.03–2.27)	0.003†
Ht	0.34 (0.25–0.40)	0.31 (0.25–0.35)	0.32 (0.29–0.35)	0.484
Uric acid (S), mg/dL	4.9 (3.8–5.7)	5.9 (4.3–8.3)	7.4 (6.1–9.2)	0.141
Creatinine (S), mg/dL	0.81 (0.67–1.16)	0.94 (0.66–2.69)	1.31 (0.84–1.83)	0.434
Uric acid (U)	32.0 (23.8–63.3)	16.0 (9.5–27.5)	15.0 (7.3–26.8)	0.037†
Creatinine (U)	46.2 (30.4–60.5)	33.0 (22.3–57.9)	39.8 (24.3–60.6)	0.795
Na (S), mM	132 (128–134)	129 (124–133)	142 (139–144)	<0.001†
Na (U), mM	101 (75–126)	81 (42–102)	101 (66–112)	0.218
Glucose (S), mg/dL	91 (88–98)	90 (76–130)	117 (88–145)	0.205
BUN (S), mg/dL	42 (19–77)	45 (21–145)	68 (31–97)	0.696
K (S), mM	4.15 (3.88–4.56)	4.40 (3.83–4.77)	3.74 (3.42–4.09)	0.037†
GFR	91 (62–97)	79 (26–93)	46 (31–80)	0.150
Albumin (S), g/dL	33 (30–35)	33 (22–37)	34 (29–35)	0.993
AST, U/L	24 (9–28)	39 (15–85)	24 (18–32)	0.452
ALT, U/L	14 (6–22)	41 (10–72)	21 (9–28)	0.237
Total bilirubin	0.68 (0.41–1.20)	0.60 (0.40–2.12)	0.50 (0.30–0.98)	0.656
TSH	0.77 (0.60–1.73)	1.29 (0.70–2.17)	1.72 (1.31–3.31)	0.192
Cortisol	14.3 (2.3–17.2)	13.4 (8.6–18.9)	11.3 (8.0–18.2)	0.799

BMI, body mass index; Ht, hematocrit; BUN, blood urea nitrogen; S, serum; U, urinary; GFR, glomerular filtration rate; AST, aspartate aminotransferase; ALT, alanine aminotransferase; TSH, thyroid stimulating hormone; COPD, chronic obstructive pulmonary disease; † Statistically significant at p < 0.05.

## Discussion

This is the first study in the literature, to the best of our knowledge, in which urinary urodilatin levels were measured in patients with RSW. On a preliminary basis, we can note how this study confirms the data that has already emerged from the literature which maintains that RSW is a cause of hyponatremia even more common than SIADH (we recruited 23 patients with hyponatremia, of which 6 patients met the diagnostic criteria of RSW and 2 had a SIADH). (Maesaka et al., 2018; Musch and Decaux, 2019). We also observed how patients with RSW display significantly higher mean urodilatin levels than both patients with (median 5.46 vs. 0.57 ng/mL, p = 0.006) or without hyponatremia (median 5.46 vs. 0.27 ng/mL, p < 0.001) (Figure 1). Statistically significant higher mean levels of urodilatin were also observed when patients with RSW were compared with the other two groups of patients considered together (5.46 vs. 0.32 ng/mL, MW test p < 0.001). This suggests a possible role of urodilatin in the pathogenesis of RSW. Indeed the statistically significant increase in the levels of this molecule in patients with RSW was found not only against the subjects without hyponatremia, but also against the subjects with hyponatremia from other causes. This data leads us to believe that the increase in urinary urodilatin levels is quite peculiar to subjects with RSW, and that this could be the cause of hyponatremia in these subjects, while several other mechanisms are involved in the genesis of hyponatremia from causes other than RSW. The potential role of urodilatin in RSW is supported by the lack of significant differences in proANP levels across the three groups (overall KS test p = 0.266) or between RSW patients and controls with or without hyponatremia (4.9 vs. 9.7 nM, MW test p = 0.122). Therefore, as other studies in the literature have shown, circulating natriuretic peptides such as ANP do not seem to play a role in this syndrome (Cerdà-Esteve et al., 2008). Another data supporting this issue highlighted in this study is that, in the group of patients with RSW, the levels of proANP are inversely related to the urinary output of 24 h (rho = −0.975, p = 0.005). This suggests that the main cause of massive diuresis in these patients is urodilatin, while the circulating ANP levels are instead reduced due to a probable negative feedback mechanism put in place to try to contain the renal loss of water and sodium in these patients. This consideration is also supported by the work of Youmans et al. who found *in vitro* natriuretic activity in urine of neurosurgical patients with RSW (Youmans et al., 2013). The main diagnostic challenge of RSW lies in the distinction between RSW and SIADH. In this regard, the gold standard for the determination of the extracellular volume is represented by the dilution methods with radioisotopes (e.g., techniques using red blood cells labeled with radiochromate or albumin labeled with radioiodine) as they are reliable and highly reproducible (Recommended methods for measurement of, 1980; Nelson et al., 1981; Wijdicks et al., 1985; Sivakumar et al., 1994). However, these methods are of little use in clinical practice as they do not give immediate results, are rather complex to perform and have a high cost. An alternative method for the distinction between RSW and SIADH has recently been proposed, represented by the dosage of the excreted fraction of urate (Fractional Excretion urate, FEurate) (Assadi and Mazaheri, 2020; Musch and Decaux, 2019; Maesaka et al., 2014). Maesaka et al. observed that both in SIADH and RSW we will find a high FEurate (>11%). With the correction of blood sodium, the FEurate will tend to normalize in the SIADH (4%–11%) while it will remain >11% in the RSW.^51^ However, this method may not be easy to apply in clinical practice. In fact, the need to calculate the FEurate after correction of natremia values can collide with the difficulties of clinical management of acutely ill patients. It can be difficult to bring natremia back to the normal range in good time, and it may be necessary for various reasons to introduce drugs into therapy that could interfere with urate excretion and therefore with the calculation of FEurate. Furthermore, this method has not been validated by other authors. For these reasons it was not used in the present study. The above mentioned important difference in urinary urodilatin levels could be very useful in the diagnostic setting and can help overcome the diagnostic approaches used up to now. The analysis of the ROC curves (Figure 2) showed an area under the curve (AUC) of 0.94 (95% CI 0.86–1.00), with the best cut-off as a urodilatin value of 2.87 ng/mL. This value corresponds to a sensitivity of 1.00, specificity of 0.88, positive predictive value of 0.60 and negative of 1.00. These data suggest a strong diagnostic power of urodilatin and, if confirmed by further studies, it could be a breakthrough in clinical management and research concerning RSW. It would be the first finding of a diagnostic marker that would greatly facilitate not only the differential diagnosis with SIADH, but the recognition of RSW in general. In fact, as we have seen, the statistically significant increase of urinary urodilatin levels in patients with RSW was found not only against patients without hyponatremia, but also against those with hyponatremia from other causes. This confers a particular sensitivity of the urodilatin towards RSW diagnosis. A diagnostic marker for RSW would allow to overcome the problems of the diagnostic approaches adopted up to now, namely,: the need to have an accurate and reliable evaluation of the ECV; the need to calculate the FEurate twice, the second time after correction of the blood sodium.

### Limitations

**FIGURE 3:**
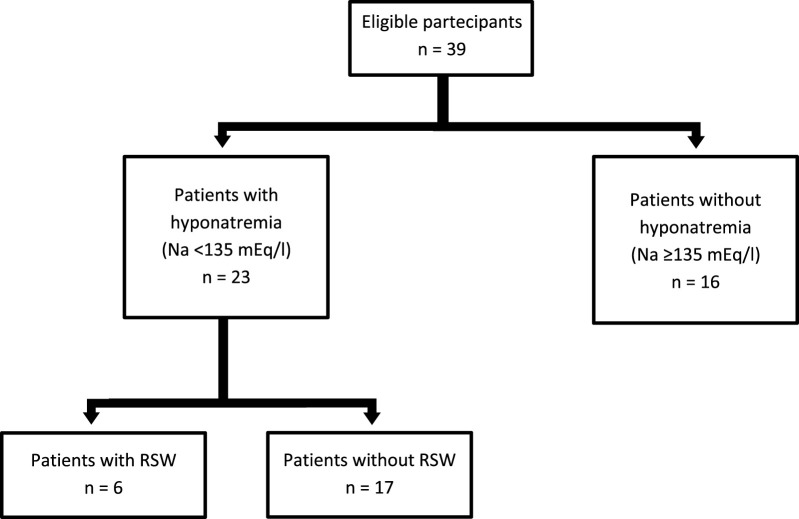
STARD diagram.

The possible limitations of this study must be considered, first of all the small number of recruited patients with RSW (Figure 3). This was due both to the fact that RSW is a relatively infrequent syndrome, and to the use of very restrictive criteria for the diagnosis of RSW. On the other hand, the use of these diagnostic criteria has allowed us to make an accurate differential diagnosis between SIADH and RSW. It is important to emphasize that all other studies in the literature on natriuretic peptide measurement in RSW have included populations of fewer than 10 individuals. Therefore, the size of our population is consistent with previous studies and may be sufficient to provide preliminary results on urinary urodilatin measurement in this context, encouraging further research. It must be pointed out that the kit used for the urodilatin dosage does not distinguish urodilatin from ANP, and this could cast doubts on the specificity of the urinary urodilatin dosage which could be altered by the presence of ANP in the urine. However, it should be considered that ANP is rapidly degraded in the renal tubule as opposed to urodilatin which is instead resistant to this degradation, and that the use of specific kits in the past has shown that almost all of the natriuretic peptides that can be measured in urine is represented precisely by urodilatin (Zaid et al., 2007; Drummer et al., 1991; Feller et al., 1989). Furthermore, the statistical analysis did not show any correlation between the circulating levels of proANP and urinary urodilatin levels in the entire population studied (Speaman’s rho = 0.059, p = 0.739). It must also be considered that, having included in the group of cases with RSW only patients with polyuria that cannot be explained by the causes listed in Table 2, it is possible that we have selected only a subpopulation of patients with RSW in the “hyperacute” phase. In fact, we know that RSW can be an insidious syndrome, with an oscillating trend and with effects on natremia and diuresis that are not always so pronounced (Maesaka et al., 2020; Della Corte et al., 2018; Musch and Decaux, 2019). Therefore it must be clarified whether urodilatin secretion remains significantly elevated even in patients with a “moderate” RSW, or whether it is instead a characteristic of patients with RSW in the “acute” phase. Further studies will certainly help us resolve this question as well.

## Conclusion

This case-control study provides novel insights into urinary urodilatin levels in RSW patients, with potential implications for understanding the syndrome’s pathogenesis, refining diagnostic criteria, and improving clinical management. Further studies will be needed to confirm our findings by expanding the study population, including cases of chronic RSW, and utilizing an external validation cohort. We hope that further scientific research will provide greater insight into this fascinating topic.

## Clinical significance

Renal Salt Wasting Syndrome (RSW) is a clinical syndrome with laboratory characteristics completely overlapping with the syndrome of inappropriate antidiuretic hormone secretion (SIADH). The differential diagnosis is still difficult, and the pathogenesis of this syndrome has not been clarified. In this case-control study, urinary urodilatin levels in patients with RSW were found up to 10 times higher than in controls, the analysis of ROC curves showed good diagnostic power of urinary urodilatin toward RSW diagnosis. Urinary urodilatin assay could be potentially used as a new diagnostic marker of Renal Salt Wasting Syndrome. Furthermore, these data may provide important clues to the underlying pathogenesis of RSW.

## Data Availability

The original contributions presented in the study are included in the article/supplementary material, further inquiries can be directed to the corresponding author.

## References

[B1] Anker S. D. Ponikowski P. Mitrovic V. Peacock W. F. Filippatos G. (2015). Ularitide for the treatment of acute decompensated heart failure: from preclinical to clinical studies. Eur. Heart J. 36 (12), 715–723. Epub 2015 Feb 10. PMID: 25670819; PMCID: PMC4368857. 10.1093/eurheartj/ehu484 25670819 PMC4368857

[B2] Askar A. Tarif N. (2007). Cerebral salt wasting in a patient with head trauma: management with saline hydration and fludrocortisone. Saudi J. Kidney Dis. Transpl. 18 (1), 95–99. PMID: 17237900. 17237900

[B3] Assadi F. Mazaheri M. (2020). Differentiating syndrome of inappropriate ADH, reset osmostat, cerebral/renal salt wasting using fractional urate excretion. J. Pediatr. Endocrinol. Metab. 34 (1), 137–140. PMID: 33180045. 10.1515/jpem-2020-0379 33180045

[B4] Berendes E. Walter M. Cullen P. Prien T. Van Aken H. Horsthemke J. (1997). Secretion of brain natriuretic peptide in patients with aneurysmal subarachnoid haemorrhage. Lancet 349 (9047), 245–249. PMID: 9014912. 10.1016/s0140-6736(96)08093-2 9014912

[B5] Bitew S. Imbriano L. Miyawaki N. Fishbane S. Maesaka J. K. (2009). More on renal salt wasting without cerebral disease: response to saline infusion. Clin. J. Am. Soc. Nephrol. 4 (2), 309–315. PMID: 19201917; PMCID: PMC2637602. 10.2215/CJN.02740608 19201917 PMC2637602

[B6] Borgmann H. (2014). “Polyuria,” in Urology at a glance. Editors Merseburger A. Kuczyk M. Moul J. (Berlin, Heidelberg: Springer). 10.1007/978-3-642-54859-8_5

[B7] Cerdà-Esteve M. Cuadrado-Godia E. Chillaron J. J. Pont-Sunyer C. Cucurella G. Fernández M. (2008). Cerebral salt wasting syndrome: review. Eur. J. Intern Med. 19 (4), 249–254. Epub 2008 Mar 7. PMID: 18471672. 10.1016/j.ejim.2007.06.019 18471672

[B8] Costa K. N. Nakamura H. M. Cruz L. R. Miranda L. S. V. F. d. Santos-Neto R. C. d. Cosme S. d. L. (2009). Hyponatremia and brain injury: absence of alterations of serum brain natriuretic peptide and vasopressin. Arq. Neuropsiquiatr. 67 (4), 1037–1044. PMID: 20069215. 10.1590/s0004-282x2009000600014 20069215

[B9] Della Corte V. Pacinella G. Todaro F. Pecoraro R. Tuttolomondo A. (2023). The natriuretic peptide system: a single entity, pleiotropic effects. Int. J. Mol. Sci. 24 (11), 9642. PMID: 37298592; PMCID: PMC10253422. 10.3390/ijms24119642 37298592 PMC10253422

[B10] Della Corte V. Tuttolomondo A. Pecoraro R. Pinto A. (2018). Chronic hyponatremia in a patient with renal salt wasting and without cerebral disease: relationship between RSW, risk of fractures and cognitive impairment. Intern Emerg. Med. 13 (8), 1167–1171. Epub 2018 Aug 13. PMID: 30105494. 10.1007/s11739-018-1926-7 30105494

[B11] DiBona G. F. (2000). Neural control of the kidney: functionally specific renal sympathetic nerve fibers. Am. J. Physiol. Regul. Integr. Comp. Physiol. 279 (5), R1517–R1524. PMID: 11049831. 10.1152/ajpregu.2000.279.5.R1517 11049831

[B12] Drummer C. Fiedler F. Bub A. Kleefeld D. Dimitriades E. Gerzer R. (1993). Development and application of a urodilatin (CDD/ANP-95-126)-specific radioimmunoassay. Pflugers Arch. 423 (5-6), 372–377. PMID: 8351194. 10.1007/BF00374930 8351194

[B13] Drummer C. Fiedler F. König A. Gerzer R. (1991). Urodilatin, a kidney-derived natriuretic factor, is excreted with a circadian rhythm and is stimulated by saline infusion in man. J. Am. Soc. Nephrol. 1 (9), 1109–1113. 10.1681/ASN.V191109 1832983

[B14] Drummer C. Franck W. Heer M. Forssmann W. G. Gerzer R. Goetz K. (1996). Postprandial natriuresis in humans: further evidence that urodilatin, not ANP, modulates sodium excretion. Am. J. Physiol. 270 (2 Pt 2), F301–F310. PMID: 8779891. 10.1152/ajprenal.1996.270.2.F301 8779891

[B15] Emmeluth C. Drummer C. Gerzer R. Bie P. (1992). Roles of cephalic Na+ concentration and urodilatin in control of renal Na+ excretion. Am. J. Physiol. 262 (3 Pt 2), F513–F516. PMID: 1313647. 10.1152/ajprenal.1992.262.3.F513 1313647

[B16] Feller S. M. Gagelmann M. Forssmann W. G. (1989). Urodilatin: a newly described member of the ANP family. Trends Pharmacol. Sci. 10 (3), 93–94. PMID: 2531950. 10.1016/0165-6147(89)90199-5 2531950

[B17] Forssmann W. G. Richter R. Meyer M. (1998). The endocrine heart and natriuretic peptides: histochemistry, cell biology, and functional aspects of the renal urodilatin system. Histochem Cell Biol. 110 (4), 335–357. PMID: 9792413. 10.1007/s004180050295 9792413

[B18] Forte L. R. Jr (2004). Uroguanylin and guanylin peptides: pharmacology and experimental therapeutics. Pharmacol. Ther. 104 (2), 137–162. PMID: 15518884. 10.1016/j.pharmthera.2004.08.007 15518884

[B19] Fukuoka T. Tsurumi Y. Tsurumi A. (2017). Cerebral salt-wasting syndrome caused by minor head injury. Case Rep. Emerg. Med. 2017, 8692017. Epub 2017 Jan 17. PMID: 28194285; PMCID: PMC5282430. 10.1155/2017/8692017 28194285 PMC5282430

[B20] Gao Y. L. Xin H. N. Feng Y. Fan J. W. (2006). Human plasma DNP level after severe brain injury. Chin. J. Traumatol. 9 (4), 223–227. PMID: 16848994. 16848994

[B21] Goetz K. Drummer C. Zhu J. L. Leadley R. Fiedler F. Gerzer R. (1990). Evidence that urodilatin, rather than ANP, regulates renal sodium excretion. J. Am. Soc. Nephrol. 1 (6), 867–874. PMID: 1966524. 10.1681/ASN.V16867 1966524

[B22] Herten M. Lenz W. Gerzer R. Drummer C. (1998). The renal natriuretic peptide urodilatin is present in human kidney. Nephrol. Dial. Transpl. 13 (10), 2529–2535. PMID: 9794555. 10.1093/ndt/13.10.2529 9794555

[B23] Hirsch J. R. Meyer M. Forssmann W. G. (2006). ANP and urodilatin: who is who in the kidney. Eur. J. Med. Res. 11 (10), 447–454. PMID: 17107879. 17107879

[B24] Kurokawa Y. Uede T. Ishiguro M. Honda O. Honmou O. Kato T. (1996). Pathogenesis of hyponatremia following subarachnoid hemorrhage due to ruptured cerebral aneurysm. Surg. Neurol. 46 (5), 500–508. PMID: 8874554. 10.1016/s0090-3019(96)00034-1 8874554

[B25] Lenz W. Herten M. Gerzer R. Drummer C. (1999). Regulation of natriuretic peptide (urodilatin) release in a human kidney cell line. Kidney Int. 55 (1), 91–99. PMID: 9893117. 10.1046/j.1523-1755.1999.00242.x 9893117

[B26] Leonard J. Garrett R. E. Salottolo K. Slone D. S. Mains C. W. Carrick M. M. (2015). Cerebral salt wasting after traumatic brain injury: a review of the literature. Scand. J. Trauma Resusc. Emerg. Med. 23, 98. PMID: 26561391; PMCID: PMC4642664. 10.1186/s13049-015-0180-5 26561391 PMC4642664

[B27] Levey A. S. Stevens L. A. (2010). Estimating GFR using the CKD Epidemiology Collaboration (CKD-EPI) creatinine equation: more accurate GFR estimates, lower CKD prevalence estimates, and better risk predictions. Am. J. Kidney Dis. 55 (4), 622–627. PMID: 20338463; PMCID: PMC2846308. 10.1053/j.ajkd.2010.02.337 20338463 PMC2846308

[B28] Lisy O. Jougasaki M. Heublein D. M. Schirger J. A. Chen H. H. Wennberg P. W. (1999). Renal actions of synthetic dendroaspis natriuretic peptide. Kidney Int. 56 (2), 502–508. PMID: 10432389. 10.1046/j.1523-1755.1999.00573.x 10432389

[B29] Maesaka J. K. Gupta S. Fishbane S. (1999). Cerebral salt-wasting syndrome: does it exist? Nephron 82 (2), 100–109. PMID: 10364700. 10.1159/000045384 10364700

[B30] Maesaka J. K. Imbriano L. Mattana J. Gallagher D. Bade N. Sharif S. (2014). Differentiating SIADH from cerebral/renal salt wasting: failure of the volume approach and need for a new approach to hyponatremia. J. Clin. Med. 3 (4), 1373–1385. PMID: 26237607; PMCID: PMC4470189. 10.3390/jcm3041373 26237607 PMC4470189

[B31] Maesaka J. K. Imbriano L. J. Ali N. M. Ilamathi E. (2009). Is it cerebral or renal salt wasting? Kidney Int. 76 (9), 934–938. Epub 2009 Jul 29. PMID: 19641485. 10.1038/ki.2009.263 19641485

[B32] Maesaka J. K. Imbriano L. J. Miyawaki N. (2018). High prevalence of renal salt wasting without cerebral disease as cause of hyponatremia in general medical wards. Am. J. Med. Sci. 356 (1), 15–22. Epub 2018 Apr 7. PMID: 30049325. 10.1016/j.amjms.2018.03.020 30049325

[B33] Maesaka J. K. Imbriano L. J. Miyawaki N. (2020). Evolution and evolving resolution of controversy over existence and prevalence of cerebral/renal salt wasting. Curr. Opin. Nephrol. Hypertens. 29 (2), 213–220. PMID: 31904619. 10.1097/MNH.0000000000000592 31904619

[B34] Maesaka J. K. Imbriano L. J. Pinkhasov A. Muralidharan R. Song X. Russo L. M. (2021). Identification of a novel natriuretic protein in patients with cerebral-renal salt wasting-implications for enhanced diagnosis. Am. J. Med. Sci. 361 (2), 261–268. Epub 2020 Jul 13. PMID: 33526214. 10.1016/j.amjms.2020.07.015 33526214

[B35] Maesaka J. K. Miyawaki N. Palaia T. Fishbane S. Durham J. H. (2007). Renal salt wasting without cerebral disease: diagnostic value of urate determinations in hyponatremia. Kidney Int. 71 (8), 822–826. Epub 2007 Feb 21. PMID: 17311074. 10.1038/sj.ki.5002093 17311074

[B36] McGirt M. J. Blessing R. Nimjee S. M. Friedman A. H. Alexander M. J. Laskowitz D. T. (2004). Correlation of serum brain natriuretic peptide with hyponatremia and delayed ischemic neurological deficits after subarachnoid hemorrhage. Neurosurgery 54 (6), 1369–1374. PMID: 15157293. 10.1227/01.neu.0000125016.37332.50 15157293

[B37] Meyer M. Richter R. Brunkhorst R. Wrenger E. Schulz-Knappe P. Kist A. (1996). Urodilatin is involved in sodium homeostasis and exerts sodium-state-dependent natriuretic and diuretic effects. Am. J. Physiol. 271 (3 Pt 2), F489–F497. PMID: 8853410. 10.1152/ajprenal.1996.271.3.F489 8853410

[B38] Musch W. Decaux G. (2019). Hyponatremia secondary to transient renal salt wasting (TRSW): a not so uncommon observation in the elderly. Clin. Nephrol. 91 (6), 344–352. PMID: 30935460. 10.5414/CN109472 30935460

[B39] Nelson P. B. Seif S. M. Maroon J. C. Robinson A. G. (1981). Hyponatremia in intracranial disease: perhaps not the syndrome of inappropriate secretion of antidiuretic hormone (SIADH). J. Neurosurg. 55 (6), 938–941. PMID: 7299468. 10.3171/jns.1981.55.6.0938 7299468

[B40] Oh J. Y. Shin J. I. (2015). Syndrome of inappropriate antidiuretic hormone secretion and cerebral/renal salt wasting syndrome: similarities and differences. Front. Pediatr. 2, 146. PMID: 25657991; PMCID: PMC4302789. 10.3389/fped.2014.00146 25657991 PMC4302789

[B41] Oh M. S. Carroll H. J. (1999). Cerebral salt-wasting syndrome. We need better proof of its existence. Nephron 82 (2), 110–114. PMID: 10364701. 10.1159/000045385 10364701

[B42] Peters J. P. Welt L. G. Sims E. A. Orloff J. Needham J. (1950). A salt-wasting syndrome associated with cerebral disease. Trans. Assoc. Am. Physicians 63, 57–64. PMID: 14855556. 14855556

[B43] Potter L. R. Yoder A. R. Flora D. R. Antos L. K. Dickey D. M. (2009). Natriuretic peptides: their structures, receptors, physiologic functions and therapeutic applications. Handb. Exp. Pharmacol. (191), 341–366. PMID: 19089336; PMCID: PMC4855512. 10.1007/978-3-540-68964-5_15 19089336 PMC4855512

[B44] Recommended methods for measurement of red-cell and plasma volume: international Committee for Standardization in Haematology (1980). J. Nucl. Med.;21(8):793–800. PMID: 7400838. 7400838

[B45] Schwartz W. B. Bennett W. Curelop S. Bartter F. C. (1957). A syndrome of renal sodium loss and hyponatremia probably resulting from inappropriate secretion of antidiuretic hormone. Am. J. Med. 23 (4), 529–542. PMID: 13469824. 10.1016/0002-9343(57)90224-3 13469824

[B46] Singh S. Bohn D. Carlotti A. P. Cusimano M. Rutka J. T. Halperin M. L. (2002). Cerebral salt wasting: truths, fallacies, theories, and challenges. Crit. Care Med. 30 (11), 2575–2579. PMID: 12441772. 10.1097/00003246-200211000-00028 12441772

[B47] Sivakumar V. Rajshekhar V. Chandy M. J. (1994). Management of neurosurgical patients with hyponatremia and natriuresis. Neurosurgery 34 (2), 269–274. PMID: 8177388. 10.1227/00006123-199402000-00010 8177388

[B48] Tomida M. Muraki M. Uemura K. Yamasaki K. (1998). Plasma concentrations of brain natriuretic peptide in patients with subarachnoid hemorrhage. Stroke 29 (8), 1584–1587. PMID: 9707197. 10.1161/01.str.29.8.1584 9707197

[B49] Tsubokawa T. Kurita H. Kaneko N. Iino N. Shiokawa Y. (2003). Clinical significance of natriuretic peptides in patients with aneurysmal subarachnoid hemorrhage. No To Shinkei 55 (11), 953–960. Japanese. PMID: 14727535. 14727535

[B50] Wijdicks E. F. Vermeulen M. ten Haaf J. A. Hijdra A. Bakker W. H. van Gijn J. (1985). Volume depletion and natriuresis in patients with a ruptured intracranial aneurysm. Ann. Neurol. 18 (2), 211–216. 10.1002/ana.410180208 4037761

[B51] Youmans S. J. Fein M. R. Wirkowski E. Maesaka J. K. (2013). Demonstration of natriuretic activity in urine of neurosurgical patients with renal salt wasting. F1000Res 2, 126. PMID: 24358843; PMCID: PMC3752684. 10.12688/f1000research.2-126.v2 24358843 PMC3752684

[B52] Zaid A. Eliahu G. Klein H. Keiser Harry (2007). Urodilatin: a natriuretic peptide of renal origin. Cardiovasc. Drug Rev. 10, 199–210. 10.1111/j.1527-3466.1992.tb00246.x

[B53] Zhang W. Li S. Visocchi M. Wang X. Jiang J. (2010). Clinical analysis of hyponatremia in acute craniocerebral injury. J. Emerg. Med. 39 (2), 151–157. Epub 2008 Aug 23. PMID: 18722740. 10.1016/j.jemermed.2008.01.027 18722740

